# Supplementary material for: Evolutionary analysis of vision genes identifies potential drivers of visual differences between giraffe and okapi
Source: PeerJ. 2017 Apr 6;5:e3145. doi: 10.7717/peerj.3145 (PMC5385128; doi:10.7717/peerj.3145)
Supplement: File S1 [file peerj-05-3145-s001.docx]

**Candidate vision genes in mammals and their actual/possible role in vision**

| **Gene name** | **Gene function** | **Vision Phenotypes** | **Sample References** |
| --- | --- | --- | --- |
| GNGT1 | The transducin gamma subunit of G-protein is required for the GTPase activity, mediating rhodopsin-effector interaction in phototransduction signaling system. | Null mice for the protein exhibit reduced signal amplification leading to rod visual sensitivity; | Lobanova et al. 2008; Kolesnikov et al. 2011; |
| RS1 | Secreted from photoreceptor cells of the outer retina and bipolar cells  of the inner retina. The protein plays a crucial role in the cellular stabilization and organization of the retina | Responsible for X-linked retinoschisis (XLRS), a retinal dystrophy that leads to schisis (splitting) of the neural retina. Leads to reduced visual acuity in affected men. | Skorczyk & Krawczyński 2012; |
| OPN1SW | Short wavelength sensitive cone pigment, a protein that changes its conformation following isomerization of 11-*cis*-retinal into all-*trans*-retinal by light in the blue region of spectrum which activates its binding to G-protein transducin triggering phototransduction cascade. | Mutations in the protein leads to color blindness and color blindness, tritan | Nathans et al. 1986; Weitz et al. 1992; Chang et al.1995 |
| CNGA2 | Membrane ion channel which mediate phototransduction in photoreceptors and chemotransduction in olfactory neurons | Highly expressed in photoreceptors | Pifferi et al. 2006;Nache et al. 2013; |
| CNGA4 | Membrane ion channel which mediate phototransduction in photoreceptors and chemotransduction in olfactory neurons | Highly expressed in photoreceptors | Pifferi et al. 2006; Nache et al. 2013 |
| SLC4A7 | Sodium- and bicarbonate-dependent cotransporter maintain normal p^H^ homeostasis in photoreceptor and auditory cells | Mice null for the gene show progressive retinal degeneration characterized by selective loss of photoreceptor cells | Pushkin et al. 1999; Bok et al. 2003; |
| GUCA1A | Stimulates guanylyl cyclase 1 (GC1) when free calcium ions concentration is low and inhibits GC1 when free calcium ions concentration is elevated. | Ca2+-sensitive regulation of GC1 is a key event in recovery of the dark state of rod photoreceptors following light exposure; mutations in the gene are linked to autosomal dominant cone dystrophy, a disease characterized by reduced visual acuity. | Payne et al. 1998; Li et al. 2001; |
| CRYAA | Structural proteins in the lens fiber cells which contributes to the transparency and refractive index of the lens; its chaperone-like activity binds and prevent aggregation of unfolded or denatured proteins. | Defects in the gene cause autosomal dominant congenital cataract | Litt et al. 1998; Horwitz 2003; Nagaraj et al. 2012; |
| GNAT1 | The transducin alpha subunit of G-protein in rod cells is required for the GTPase activity, mediating rhodopsin-effector interaction during phototransduction cascade. | Mutations in the gene are associated with congenital stationary night blindness. | Dryja et al. 1996; Goc et al. 2009; Naeem et al. 2012; |
| PLCB4 | Catalyze the reaction that produces second messengers diacylglycerol (DAG) and inositol trisphosphate (IP3) | Mutant mice null for the gene show diminished visual response suggesting the gene's role visual signal processing. | Lee et al. 1994; Jiang et al. 1996; Lee et al. 1994; Jiang et al. 1996; |
| PDE6D | Following activation by transducin, the enzyme hydrolyze cGMP, a key messenger molecule in phototransduction | Mutations in a gene causes reduced enzyme activity leading to retinal degeneration in mice | Deterre et al. 1988; Bowes et al. 1990; |
| SAG | Photoreceptor regulation; binds to photoactivated-phosphorylated rhodopsin, apparently to desensitize rhodopsin which prevent transducin-mediated activation of phosphodiesterase | Mutation in the gene causes Oguchi disease characterized by discoloration of the fundus and retininis pigmentosa | Kuhn et al. 1984; Fuchs et al. 1995; Nakazawa et al. 1998; |
| GNB1 | The transducin beta subunit of a G-protein required for the GTPase activity, mediating rhodopsin-effector interaction in phototransduction signaling system. | Disruptions of the gene leads to retinal degeneration | Kitamura et al. 2006; Chang 2013; |
| PRPH2 | An integral membrane glycoprotein that is present in the rims of photoreceptor  outer segment disks; stabilize the disk rim through heterophilic interactions  with the related nonglycosylated protein rom1 | Mutations in the gene leads to various retinal degenarations including retininis pigmentosa, pattern dystrophy and macular degerations | Connell et al. 1991; Kajiwara et al. 1991; Jacobson et al. 1996; |
| PDC | May regulate visual phototransduction or integrity of photoreceptor metabolism | Abundantly expressed in the retina | Zhu & Craft 2000; Nishiguchi et al. 2004; |
| RPGRIP1 | a key component of cone and rod photoreceptor cells that interacts with retinitis pigmentosa GTPase regulator protein | Retininis pigmentosa and cone-rod dystrophy | Roepman et al. 2000; Kuznetsova et al. 2012; |
| ARR3 | May play a role in regulating opsin functions through interacting with photoactivated-phosphorylated red/green opsins. | Abundantly expressed in the retina | Craft et al. 1994; Gurevich et al. 1995; |
| ACCN1 | Proton-gated ion channels thought to modulate neuronal excitability through pH sensing | Abundantly expressed in photoreceptors and may confer retinal protection against light | Ettaiche et al. 2004; Lingueglia 2007; |
| OPN1LW | Long-wavelength sensitive opsin, a protein that changes its conformation following isomerization of 11-*cis*-retinal into all-*trans*-retinal by light in the red region of spectrum which activates its binding to G-protein transducin triggering phototransduction cascade | Mutations in the protein leads to blue-cone monochromacy | Nathans et al, 1986; Nathans et al. 1993; |
| CDS2 | Regulates availability of second messengers in GPCR pathways. | Highly expressed in the retina | Volta et al. 1999; |
| TTR | A carrier protein that transports thyroid hormones in the plasma and cerebrospinal fluid, and also transports retinol (vitamin A) in the plasma. | Highly expressed in the retina; mutations in the gene may disrupt the availability of retinal in the eye | Bernis et al. 1994; Bui et al. 2001; |
| OPN4 | Similar to other opsins but its photosensitivity is limited to pupillar reflex, circardian rythms, and other non-image forming responses to light. | Expressed in the retina ganglion cells; mice exhibit a shorter than normal period when exposed to constant light | Provencio et al. 2002; Tu et al. 2005; Panda et al. 2002; Tu et al. 2005; |
| PCP2 | Functions as a cell-type specific modulator for G protein-mediated cell signaling inhibiting the dissiciation of GDP from alpha subunit of G-protein. | Expressed in the retinal ON bipolar cells; maintain the hyperpolarization of cell and speeds up visual response | Guan et al. 2005; Xu et al. 2008; |
| LUM | Structural function forming the bulk of corneal connective tissue | Null mice for the gene leads to opaque cornea suggesting the role of the protein in cornea light transparency | Chakravarti et al. 2000; Chakravarti et al. 2003; |
| AIPL1 | Interacts (chaperone activity) with the visual effector enzyme phosphodiesterase-6 | Mutations cause Leber congenital amaurosis, a severe, early onset, inherited retinopathy | Sohocki et al. 2003; Majumder et al. 2013; |
| RDH12 | Catalytical role in the metabolism of retinoids, chromophores involved in vision; may be involved in the formation of 11-cis-retinal from 11-cis-retinol during regeneration of the cone visual pigments | Mutations cause Leber congenital amaurosis, a severe form inherted retinal dystrophy | Haeseleer et al. 2002; Perrault et al. 2004; |
| RPE65 | Critical in the visual retinoid cycle; the production of 11-cis retinal and in visual pigment regeneration | Mutations causes severe retinal dystrophy | Gu et al. 1997; Moiseyev et al. 2005; |
| RDH11 | NADPH-dependent retinal reductase converting all-trans-retinol to all-trans-retinal | Expressed in the retina; gene disruption in mice exhibit delayed dark adaptation | Haeseleer et al. 2002; Kasus-Jacobi et al. 2005; |
| GNAT2 | Transducin alpha subunit of G-protein coupled to cone visual pigment required for GTPase activity, mediating photopsin-effector interaction in phototransduction signaling system | Mutations in the gene result in achromatopsia, failure to discriminate colors in human | Aligianis et al. 2002; Kohl et al. 2002; |
| RHO | Primary visual pigment in retinal rod cells; initiates the visual transduction cascade following photo-excitation; very sensitive to light enabling dark vision | Mutations in the gene causes autosomal dominant retinitis pigmentosa and night blindness | McIness & Bascom 1992; Sieving et al. 1995; |
| CYP27B1 | Catalyzes the conversion of 25-hydroxyvitamin D3 (25(OH)D) to 1-alpha,25-dihydroxyvitamin D3 (1,25(OH)2D) plays an important role in normal bone growth, calcium metabolism, and tissue differentiation | Ocular barrier epithelial cells express the machinery for vitamin D3 production;an inverse association between plasma 25-hydroxyvitamin D and the presence of subretinal fibrosis was found in patients with age-related macular degeneration | Singh et al. 2013; Alsalem et al. 2014; |
| RGR | Required in the production of 11-cis-retinal by the retinal pigment epithelium (RPE) under light condition and normal regeneration of rhodopsin | Exclusively expressed in tissue adjacent to retinal photoreceptor cells, the retinal pigment epithelium and Mueller cells; mutations cause retininis pigmentosa | Morimura et al. 1999; Yang & Fong 2002; |
| RRH | RPE rhodopsin homolog; may play a role in RPE physiology either by detecting light directly or by modulating the retinoid cycle | Exclusively expressed in the RPE; mutations in the gene are implicated in various forms of retinal degenerations | Sun et al. 1997; Rivolta et al. 2006; |
| GUCA1B | Stimulates both guanylyl cyclase 1 (GC1) and guanylyl cyclase 2 (GC1) when free calcium ions concentration is low | Ca2+-sensitive regulation of GC1 is a key event in recovery of the dark state of rod photoreceptors following light exposure; mutations in the gene are linked to autosomal dominant cone dystrophy, a disease characterized by reduced visual acuity | Payne et al. 1999; Sato & Nakazawa 2004; |
| PPEF2 | May play a role in phototransduction. May dephosphorylate photoactivated rhodopsin. May function as a calcium sensing regulator of ionic currents, energy production or synaptic transmission | Expressed specifically in photoreceptors and the pineal | Sherman et al. 1997; Ramulu et al. 2001; |
| ADAMTSL4 | A member of ADAMTS (a disintegrin and metalloproteinase with thrombospondin motifs)-like gene family and encodes a protein with seven thrombospondin type 1 repeats. The thrombospondin type 1 repeat domain is found in many proteins with diverse biological functions including cellular adhesion, angiogenesis, and patterning of the developing nervous system | Wide distribution in the eye; mutation in the gene have been associated with ectopia lentis – dislocation of the lens from its optimal position | Ahram et al. 2009; Gabriel et al. 2014; |

**References**

Ahram D et al. (2009) A Homozygous Mutation in ADAMTSL4 Causes Autosomal-Recessive Isolated Ectopia Lentis. *Am. J. Hum. Gen.* *84*(2), 274–278.

Aligianis IA et al. (2002) Mapping of a novel locus for achromatopsia (ACHM4) to 1p and identification of a germline mutation in the alpha subunit of cone transducin (GNAT2). *J. Med. Genet*. *39*, 656-660.

Alsalem JA et al. (2014) Characterization of vitamin D production by human ocular barrier cells. *Inv. Opht. Vis. Sc.* *55*(4), 2140-2147.

Berni R et al. (1994) The Ile-84--> Ser amino acid substitution in transthyretin interferes with the interaction with plasma retinol-binding protein. *Journal of Biological Chemistry*, *269*(38), 23395-23398.

Bok D et al. (2003) Blindness and auditory impairment caused by loss of the sodium bicarbonate cotransporter NBC3. *Nat. Gen.* *34*(3), 313-319.

Bowes C et al. (1990) Retinal degeneration in the rd mouse is causedby a defect in the β subunit of rod cGMP-phosphodiesterase, *Nature,* 347 677–680.

Bui BV et al. (2001) Retinal anatomy and function of the transthyretin null mouse. *Exp. Eye Res.* *73*, 651–659.

Chakravarti S et al. (2000) Corneal opacity in lumican-null mice: defects in collagen fibril structure and packing in the posterior stroma. *Inv. Opht. Vis. Sc.* *41*(11), 3365-3373.

Chakravarti S et al. (2003) Ocular and scleral alterations in gene-targeted lumican-fibromodulin double-null mice. *Inv. Opht. Vis. Sc.* *44*(6), 2422-2432.

Chang BS et al. (1995) Opsin phylogeny and evolution: a model for blue shifts in wavelength regulation. *Mol. Phyl. Ev.* *4*(1), 31-43.

Chang B (2013) Mouse models for studies of retinal degeneration and diseases. *Ret. Deg. Meth. Prot.*, 27-39.

Connell G et al. (1991) Photoreceptor peripherin is the normal product of the gene responsible for retinal degeneration in the rds mouse. *Proc. Nat. Ac. Sc.* *88*(3), 723-726.

Craft CM et al. (1994) Cone arrestin identified by targeting expression of a functional family. *J. Biol. Chem*. *269*, 4613–4619.

Dryja TP et al. ( 1996) Missense mutation in the gene encoding the α subunit of rod transducin in the Nougaret form of congenital stationary night blindness. *Nat. Gen.* *13*(3), 358-360.

Ettaiche M et al. (2004) Acid-sensing ion channel 2 is important for retinal function and protects against light-induced retinal degeneration. *J. Neurosc.* *24*(5), 1005-1012.

Fuchs S et al. (1995) A homozygous 1-base pair deletion in the arrestin gene is a frequent cause of Oguchi disease in Japanese. *Nat. Gen.* *10*, 360-362.

Gabriel LA et al. (2012) ADAMTSL4, a secreted glycoprotein widely distributed in the eye, binds fibrillin-1 microfibrils and accelerates microfibril biogenesis. *Inv. Opht. Vis. Sc.* *53*(1), 461-469.

Goc A et al. (2008) Different Properties of the Native and Reconstituted Heterotrimeric G Protein Transducin†. *Biochem.*, *47*(47), 12409-12419.

Gu SM et al. (1997) Mutations in RPE65 cause autosomal recessive childhood-onset severe retinal dystrophy. *Nat. Genet. 17*, 194–197.

Guan J & Denker BM (2005) Purkinje cell protein-2 (Pcp2) stimulates differentiation in PC12 cells by Gβγ-mediated activation of Ras and p38 MAPK. *Biochem. Journ.* *392*(2), 389-397.

Gurevich VV et al. (1995) Arrestin Interactions with G Protein-coupled Receptors DIRECT BINDING STUDIES OF WILD TYPE AND MUTANT ARRESTINS WITH RHODOPSIN, β2-ADRENERGIC, AND m2 MUSCARINIC CHOLINERGIC RECEPTORS. *J. Biol. Chem.* ***270****(2)*, 720-731.

Haeseleer F et al. (2002) Dual-substrate specificity short chain retinol dehydrogenases from the vertebrate retina. *J. Biol. Chem. 277*, 45537–45546

Horwitz J (2003) Alpha-crystallin. *Exp. Eye Res*, *76*(2), 145-153.

Jacobson SG et al. (1996) Photoreceptor function in heterozygotes with insertion or deletion mutations in the RDS gene. *Inv. Opht. Vis. Sc.* *37*(8), 1662-1674.

Jiang H et al. (1996) Phospholipase C β4 is involved in modulating the visual response in mice. *Proc. Nat. Ac. Sc.* *93*(25), 14598-14601.

Kajiwara K et al. (1991) Mutations in the human retinal degeneration slow gene in autosomal dominant retinitis pigmentosa. *Nature*, *354*, 480-483.

Kasus-jacobi A et al. (2005) Functional characterization of mouse RDH11 as a retinol dehydrogenase involved in dark adaptation in vivo. *J. Biol. Chem.* *280*(21), 20413-20420.

Kitamura E et al. (2006) Disruption of the gene encoding the β1-subunit of transducin in the Rd4/+ mouse. *Inv. Opht. Vis. Sc.* *47*(4), 1293-1301.

Kohl S et al. (2005) CNGB3 mutations account for 50% of all cases with autosomal recessive achromatopsia. *Eur. J. Hum. Gen.* *13*(3), 302-308.

Kolesnikov AV et al. (2011) G-protein βγ-complex is crucial for efficient signal amplification in vision. *J. Neurosc.* *31*(22), 8067-8077.

Kuhn H et al. (1984) Light-induced binding of 48-kDa protein to photoreceptor membranes is highly enhanced by phosphorylation of rhodopsin. *FEBS Lett*. *176,* 473–478.

Kuznetsova T et al. (2012) Exclusion of RPGRIP1 ins44 from Primary Causal Association with Early-Onset Cone–Rod Dystrophy in Dogs Cone–Rod Dystrophy and Canine RPGRIP1. *Inv. Opht. Vis. Sc.* *53*(9), 5486-5501.

Lee CW et al. (1994) Regulation of phospholipase C-beta 4 by ribonucleotides and the alpha subunit of Gq. *J. Biol. Chem.* *269*(41), 25335-25338.

Li et al. (2001) Identification of functional regions of guanylate cyclase-activating protein 1 (GCAP1) using GCAP1/GCIP chimeras. *Biol. Chem.* *382*(8), 1179-1188.

Lingueglia E (2007) Acid-sensing ion channels in sensory perception. *J. Biol. Chem.* *282*(24), 17325-17329.

Litt M et al. (1998) Autosomal dominant congenital cataract associated with a missense mutation in the human alpha crystallin gene CRYAA. *Hum. Mol. Gen.* *7*(3), 471-474.

Lobanova ES et al. (2008) Transducin γ-subunit sets expression levels of α-and β-subunits and is crucial for rod viability. *J. Neurosc.* *28*(13), 3510-3520.

Majumder A et al. (2013) Interaction of aryl hydrocarbon receptor-interacting protein-like 1 with the farnesyl moiety. *J. Biol. Chem.* *288*(29), 21320-21328.

Makarova OV et al. (2002) Protein 61K, encoded by a gene (PRPF31) linked to autosomal dominant retinitis pigmentosa, is required for U4/U6· U5 tri‐snRNP formation and pre‐mRNA splicing. *EMBO Journ. 21*(5), 1148-1157.

McInnes RR & Bascom RA (1992) Retinal genetics: a nullifying effect for rhodopsin. *Nat. Genet*. *1*, 155-157.

Moiseyev G et al. (2005). RPE65 is the isomerohydrolase in the retinoid visual cycle. *Proc. Nat. Ac. Sc.* *102*(35), 12413-12418.

Morimura H et al. (1999) Mutations in RGR, encoding a light-sensitive opsin homologue, in patients with retinitis pigmentosa. *Nat. Genet*. *23*, 393-394.

Nache V, Eick T, Schulz E, Schmauder R, Benndorf K. 2013. Hysteresis of ligand binding in CNGA2 ion channels. *Nat. Comm*. *4*, 2866

Naeem MA et al. (2012) GNAT1 associated with autosomal recessive congenital stationary night blindness. *Inv. Opht. Vis. Sc.* *53*(3), 1353-1361.

Nagaraj RH et al. (2012) Acetylation of αA-crystallin in the human lens: Effects on structure and chaperone function. *Biochimica et Biophysica Acta (BBA)-Molecular Basis of Disease*, *1822*(2), 120-129.

Nakazawa M et al. (1998) Arrestin gene mutations in autosomal recessive retinitis pigmentosa. *Arch. Ophthal.* *116*, 498-501.

Nathans J et al. (1986) Molecular genetics of human color vision: the genes encoding blue, green, and red pigments. *Science*, *232*(4747), 193-202.

Nathans J et al. (1993) Genetic heterogeneity among blue-cone monochromats. *Am. J. Hum. Gen.* *53*(5), 987.

Nishiguchi KM et al. (2004). Mutation screening of the phosducin gene PDC in patients with retinitis pigmentosa and allied diseases. *Mol. Vis.* *10*, 62-64.

Payne AM et al. (1998) A mutation in guanylate cyclase activator 1A (GUCA1A) in an autosomal dominant cone dystrophy pedigree mapping to a new locus on chromosome 6p21. 1. *Hum. Mol. Gen.* *7*(2), 273-277.

Payne AM et al. (1999) Genetic analysis of the guanylate cyclase activator 1B (GUCA1B) gene in patients with autosomal dominant retinal dystrophies. *J. Med. Gen.* *36*(9), 691-693.

Perrault I et al. (2004) Retinal dehydrogenase 12 (RDH12) mutations in leber congenital amaurosis. *Am. J. Hum. Gen. 75*(4), 639-646.

Panda S et al. (2002) Melanopsin (Opn4) requirement for normal light-induced circadian phase shifting. *Science*, *298*(5601), 2213-2216.

Pifferi S, Boccaccio A, Menini A. 2006. Cyclic nucleotide‐gated ion channels in sensory transduction. FEBS Letters. 580(12): 2853-2859.

Pushkin A et al. (1999) Cloning, tissue distribution, genomic organization, and functional characterization of NBC3, a new member of the sodium bicarbonate cotransporter family. *J. Biol. Chem.* *274*(23), 16569-16575.

Ramulu P et al. (2001) Normal light response, photoreceptor integrity, and rhodopsin dephosphorylation in mice lacking both protein phosphatases with EF hands (PPEF-1 and PPEF-2). *Mol. Cell. Biol.* *21*(24), 8605-8614.

Rivolta C et al. (2006) Mutation screening of the peropsin gene, a retinal pigment epithelium specific rhodopsin homolog, in patients with retinitis pigmentosa and allied diseases. *Mol. Vis.* *12*, 1511-5.

Roepman R et al. (2000) The retinitis pigmentosa GTPase regulator (RPGR) interacts with novel transport-like proteins in the outer segments of rod photoreceptors. *Hum. Mol. Gen.* *9*(14), 2095-2105.

Sato M et al. (2005) Mutations in the gene coding for guanylate cyclase-activating protein 2 (GUCA1B gene) in patients with autosomal dominant retinal dystrophies. *Graefe's Arch. Clin. Exp. Opht.* *243*(3), 235-242.

Sherman et al. (1997) Identification and characterization of a conserved family of protein serine/threonine phosphatases homologous to Drosophila retinal degeneration C (rdgC). *Proc. Nat. Ac. Sci*. *94,* 11639-11644.

Sieving PA et al. (1995) Dark-light: model for nightblindness from the human rhodopsin Gly-90-->Asp mutation. *Proc. Nat. Ac. Sc. 92*, 880–884.

Singh A et al. (2013) The association between plasma 25-hydroxyvitamin D and subgroups in age-related macular degeneration: a cross-sectional study. *PloS One*, *8*(7), e70948.

Skorczyk A & Krawczyński MR. (2012) Four novel RS1 gene mutations in Polish patients with X-linked juvenile retinoschisis. *Mol. Vis.* *18*, 3004.

Sohocki MM et al. (2000) Mutations in a new photoreceptor-pineal gene on 17p cause Leber congenital amaurosis. *Nat. Gen.* *24*(1), 79-83.

Sun et al. (1997) Peropsin, a novel visual pigment-like protein located in the apical microvilli of the retinal pigment epithelium. *Proc. Nat. Ac. Sc.* *94*(18), 9893-9898.

Tu et al. (2005) Physiologic diversity and development of intrinsically photosensitive retinal ganglion cells. *Neuron*, *48*(6), 987-999.

Vithana EN et al. (2001) A human homolog of yeast pre-mRNA splicing gene, PRP31, underlies autosomal dominant retinitis pigmentosa on chromosome 19q13. 4 (RP11). *Mol. Cell*, *8*(2), 375-381.

Volta M et al. (1999) Identification and Characterization of CDS2, a Mammalian Homolog of theDrosophilaCDP-diacylglycerol Synthase Gene. *Genomics*, *55*(1), 68-77.

Weitz CJ et al. (1992) Human tritanopia associated with two amino acid substitutions in the blue-sensitive opsin. *Am. J. Hum. Gen.* *50*(3), 498.

Xu Y et al. (2008) Retinal ON bipolar cells express a new PCP2 splice variant that accelerates the light response. *J. Neurosc.* *28*(36), 8873-8884.

Yang M & Fong HK (2002) Synthesis of the all-trans-retinal chromophore of retinal G protein-coupled receptor opsin in cultured pigment epithelial cells. *J. Biol. Chem.*, *277*(5), 3318-3324.

Zhu X & Craft C M (2000) Modulation of CRX transactivation activity by phosducin isoforms. *Mol. Cell. Biol.*, *20*(14), 5216-5226.
