# Supplementary material for: Evolutionary analysis of vision genes identifies potential drivers of visual differences between giraffe and okapi
Source: PeerJ. 2017 Apr 6;5:e3145. doi: 10.7717/peerj.3145 (PMC5385128; doi:10.7717/peerj.3145)
Supplement: File S4 [file peerj-05-3145-s004.docx]

**Subset of vision genes used in the analysis of selection divergence and their comparative sequence metrics among some ruminant species**

| **Gene name** | **Functional class in vision process** | **Species** | **Percentage nucleotide identity to giraffe** | **Percentage amino acid identity to giraffe** | **RefSeq/GenBank Accession No.** |
| --- | --- | --- | --- | --- | --- |
| *Cyclic Nucleotide Gated Channel Alpha 2(CNGA2)* | Phototransduction | Okapi | 99 | 98 | NA |
|  |  | Cattle | 97 | 97 | NM_001001139.2 |
|  |  | Water buffalo | 97 | 98 | XM_006059664.1 |
|  |  | Tibetan antelope | 97 | 97 | XM_005972031.1 |
|  |  | Yak | 97 | 98 | XM_005889859.1 |
|  |  | Sheep | 97 | 94 | XM_015104729.1 |
|  |  | Goat | 97 | 97 | XM_005700487.1 |
| *Cyclic Nucleotide Gated Channel Alpha 4 (CNGA4)* | Phototransduction | Okapi | 98 | 99 | NA |
|  |  | Cattle | 97 | 97 | XM_594125.8 |
|  |  | Water buffalo | 97 | 97 | XM_006042097.1 |
|  |  | Tibetan antelope | 96 | 96 | XM_005960395.1 |
|  |  | Yak | 97 | 97 | XM_005907126.1 |
|  |  | Sheep | 97 | 96 | XM_015090403.1 |
|  |  | Goat | 97 | 93 | XM_005689771.1 |
| *Crystallin Alpha A* (*CRYAA*) | Structural property of the lens | Okapi | 98 | 100 | NA |
|  |  | Cattle | 97 | 99 | NM_174289.2 |
|  |  | Water buffalo | 97 | 99 | XM_006063827.1 |
|  |  | Tibetan antelope | 97 | 99 | XM_005956793.1 |
|  |  | Yak | 97 | 99 | XM_005907575.2 |
|  |  | Sheep | 97 | 99 | NM_001012458.1 |
|  |  | Goat | 97 | 99 | XM_005675651.2 |
| *Guanine nucleotide-binding protein G(t) subunit alpha-1* (*GNAT1*) | Phototransduction | Okapi | 99 | 100 | NA |
|  |  | Cattle | 98 | 99 | XM_010817664.2 |
|  |  | Water buffalo | 98 | 99 | XM_006053291.1 |
|  |  | Tibetan antelope | 98 | 100 | XM_005969293.1 |
|  |  | Yak | 98 | 99 | XM_005892112.1 |
|  |  | Sheep | 98 | 100 | XM_015102410.1 |
|  |  | Goat | 98 | 100 | XM_013973538.1 |
| *Guanine nucleotide-binding protein G(t) subunit alpha-2* (*GNAT2*) | Phototransduction | Okapi | 99 | 100 | NA |
|  |  | Cattle | 99 | 99 | NM_174326.2 |
|  |  | Water buffalo | 99 | 99 | XM_006052192.1 |
|  |  | Tibetan antelope | 99 | 100 | XM_005973578.1 |
|  |  | Yak | 99 | 99 | XM_005891307.1 |
|  |  | Sheep | 99 | 100 | XM_004002288.3 |
|  |  | Goat | 98 | 99 | XM_013962560.1 |
| *Guanine nucleotide-binding protein subunit beta-1* (*GNB1*) | Phototransduction | Okapi | 99 | 100 | NA |
|  |  | Cattle | 97 | 100 | XM_010813308.2 |
|  |  | Water buffalo | 98 | 100 | XM_006066888.1 |
|  |  | Tibetan antelope | 97 | 97 | XM_005969878.1 |
|  |  | Yak | 98 | 100 | XM_005888848.1 |
|  |  | Sheep | 98 | 100 | XM_004013774.3 |
|  |  | Goat | 98 | 100 | XM_005690770.2 |
| *Guanine nucleotide-binding protein* *G(t) subunit gamma-T1* (*GNGT1*) | Phototransduction | Okapi | 99 | 100 | NA |
|  |  | Cattle | 97 | 96 | NM_174327.3 |
|  |  | Water buffalo | 96 | 96 | XM_006078316.1 |
|  |  | Tibetan antelope | 97 | 97 | XM_005954515.1 |
|  |  | Yak | 97 | 96 | XM_005909692.1 |
|  |  | Sheep | 97 | 97 | XM_012146569.2 |
|  |  | Goat | 97 | 97 | XM_005678931.2 |
| *Guanylate Cyclase Activator 1A* (*GUCA1A*) | Phototransduction | Okapi | 99 | 99 | NA |
|  |  | Cattle | 98 | 99 | NM_174546.2 |
|  |  | Water buffalo | 98 | 99 | XM_006069193.1 |
|  |  | Tibetan antelope | 98 | 99 | XM_005969656.1 |
|  |  | Yak | 98 | 99 | XM_014479547.1 |
|  |  | Sheep | 99 | 99 | XM_015089980.1 |
|  |  | Goat | 98 | 99 | XM_013973784.1 |
| *Guanylate Cyclase Activator 1B* (*GUCA1B*) | Phototransduction | Okapi | 99 | 100 | NA |
|  |  | Cattle | 96 | 92 | U32856.1 |
|  |  | Water buffalo | 96 | 92 | XM_006069194.1 |
|  |  | Tibetan antelope | 96 | 93 | XM_005969657.1 |
|  |  | Yak | 96 | 92 | XM_005900138.1 |
|  |  | Sheep | 96 | 93 | XM_004018819.3 |
|  |  | Goat | 97 | 93 | XM_005696310.2 |
| *Lumican* (*LUM*) | Structural property of the cornea | Okapi | 99 | 99 | NA |
|  |  | Cattle | 97 | 97 | NM_173934.1 |
|  |  | Water buffalo | 98 | 98 | XM_006062953.1 |
|  |  | Tibetan antelope | 97 | 98 | XM_005959547.1 |
|  |  | Yak | 98 | 97 | XM_014483083.1 |
|  |  | Sheep | 97 | 98 | XM_012174076.2 |
|  |  | Goat | 97 | 98 | XM_005679812.2 |
| *Long-wave-sensitive opsin-1* (*OPN1LW*) | Phototransduction | Okapi | 99 | 99 | NA |
|  |  | Cattle | 97 | 98 | NM_174566.1 |
|  |  | Water buffalo | 97 | 98 | XM_006043645.1 |
|  |  | Tibetan antelope | 96 | 98 | XM_005957735.1 |
|  |  | Yak | 97 | 98 | XM_005909080.1 |
|  |  | Sheep | 96 | 99 | XM_004022260.3 |
|  |  | Goat | 96 | 98 | XM_005701738.1 |
| *Short-wave-sensitive opsin-1* (*OPN1SW*) | Phototransduction | Okapi | 99 | 99 | NA |
|  |  | Cattle | 98 | 96 | NM_174567.1 |
|  |  | Water buffalo | 98 | 96 | XM_006074392.1 |
|  |  | Tibetan antelope | 98 | 97 | XM_005981411.1 |
|  |  | Yak | 98 | 96 | XM_005905247.1 |
|  |  | Sheep | 98 | 97 | XM_004008047.3 |
|  |  | Goat | 98 | 97 | XM_005679445.2 |
| *Phosphodiesterase subunit delta* (*PDE6D*) | Phototransduction | Okapi | 99 | 100 | NA |
|  |  | Cattle | 99 | 100 | NM_174420.2 |
|  |  | Tibetan antelope | 99 | 100 | XM_005978626.1 |
|  |  | Sheep | 99 | 100 | XM_004005001.3 |
|  |  | Goat | 99 | 100 | XM_005676672.2 |
| *Phospholipase C beta 4* (*PLCB4)* | Phototransduction | Okapi | 99 | 99 | NA |
|  |  | Cattle | 97 | 97 | NM_001166510.1 |
|  |  | Water buffalo | 98 | 99 | XM_006049745.1 |
|  |  | Tibetan antelope | 98 | 99 | XM_005956022.1 |
|  |  | Sheep | 98 | 99 | XM_004014147.3 |
|  |  | Goat | 98 | 99 | XM_013968411.1 |
| *Retinol dehydrogenase 11* (*RDH11*) | Phototransduction | Okapi | 99 | 99 | NA |
|  |  | Cattle | 97 | 97 | XM_005193708.3 |
|  |  | Water buffalo | 97 | 98 | XM_006056173.1 |
|  |  | Tibetan antelope | 97 | 99 | XM_005961538.1 |
|  |  | Yak | 97 | 97 | XM_014481441.1 |
|  |  | Sheep | 97 | 99 | XM_012181884.2 |
|  |  | Goat | 97 | 98 | XM_005686002.2 |
| *Retinol dehydrogenase 12* (*RDH12*) | Phototransduction | Okapi | 99 | 99 | NA |
|  |  | Cattle | 95 | 95 | BC142243.1 |
|  |  | Water buffalo | 95 | 95 | XM_006056172.1 |
|  |  | Tibetan antelope | 96 | 96 | XM_005961535.1 |
|  |  | Yak | 95 | 95 | XM_014481447.1 |
|  |  | Sheep | 96 | 97 | XM_004010741.3 |
|  |  | Goat | 96 | 96 | XM_005686006.1 |
| *RPE-retinal G protein-coupled receptor* (*RGR*) | Phototransduction | Okapi | 98 | 99 | NA |
|  |  | Cattle | 96 | 97 | NM_175775.2 |
|  |  | Water buffalo | 95 | 96 | XM_006072363.1 |
|  |  | Tibetan antelope | 95 | 96 | XM_005972722.1 |
|  |  | Yak | 96 | 97 | XM_005902591.2 |
|  |  | Sheep | 96 | 96 | XM_004021531.3 |
|  |  | Goat | 96 | 96 | XM_013975680.1 |
| *Rhodopsin* (*RHO*) | Phototranduction | Okapi | 99 | 99 | NA |
|  |  | Cattle | 95 | 96 | NM_001014890.2 |
|  |  | Water buffalo | 96 | 97 | XM_006078900.1 |
|  |  | Tibetan antelope | 95 | 97 | XM_005955745.1 |
|  |  | Yak | 95 | 96 | XM_005902834.1 |
|  |  | Sheep | 97 | 99 | XM_012114315.2 |
| *Retinal Pigment Epithelium-Specific Protein 65kDa* (*RPE65*) |  | Okapi | 99 | 99 | NA |
|  |  | Cattle | 99 | 99 | NM_174453.2 |
|  |  | Water buffalo | 99 | 100 | XM_006052721.1 |
|  |  | Tibetan antelope | 99 | 100 | XM_005978384.1 |
|  |  | Yak | 99 | 100 | XM_005906391.1 |
|  |  | Sheep | 99 | 100 | XM_004002070.3 |
|  |  | Goat | 99 | 100 | XM_005678276.1 |
| *S-antigen* (*SAG*) | Phototransduction | Okapi | 98 | 99 | NA |
|  |  | Cattle | 96 | 96 | XM_010803842.2 |
|  |  | Water buffalo | 96 | 97 | XM_006041760.1 |
|  |  | Tibetan antelope | 97 | 97 | XM_005962253.1 |
|  |  | Yak | 96 | 97 | XM_005895006.1 |
|  |  | Sheep | 96 | 97 | XM_015091843.1 |
|  |  | Goat | 96 | 97 | XM_005678742.1 |
